# Effect of perinatal ampicillin or amoxicillin/clavulanate exposure on maternal and infant gut microbiome, metabolome, and infant responses to the 20-valent pneumococcal conjugate vaccine

**DOI:** 10.1080/29933935.2026.2705707

**Published:** 2026-07-20

**Authors:** Emi Suzuki, Victoria Deleray, Jasmine Zemlin, Armin Kousha, Hannah Nonoguchi, Daniel Sun, Chih-Ming Tsai, Simone Zuffa, Kine Eide Kvitne, Pieter C. Dorrestein, Shirley M. Tsunoda, Victor Nizet, George Y. Liu, Fatemeh Askarian

**Affiliations:** a Department of Pediatrics, University of California San Diego, La Jolla, CA, USA; b Division of Infectious Diseases, Rady Children’s Hospital, San Diego, CA, USA; c Division of Gastroenterology, Hepatology, and Nutrition, Rady Children’s Hospital, San Diego, CA, USA; d Skaggs School of Pharmacy and Pharmaceutical Sciences, UC San Diego, La Jolla, CA, USA

**Keywords:** Perinatal antibiotic exposure, vaccine responsiveness, microbiome-metabolome interactions

## Abstract

Emerging studies suggest that antibiotics can disrupt the gut microbiome and alter vaccine-induced immune responses. However, the specific consequences of early-life exposure on neonatal immune development remain poorly understood. Here, we examined how two antibiotics frequently used in perinatal care, broad-spectrum ampicillin (AMP) and the extended-spectrum combination amoxicillin/clavulanate (AMOX/CLAV), administered during gestation and lactation, influence neonatal gut microbiome composition, fecal metabolome profiles, and responses to the 20-valent pneumococcal conjugate vaccine (PCV20). Maternal treatment with AMOX/CLAV, but not AMP, significantly reduced PCV-specific IgG titers at 4 and 6 weeks post-prime immunization compared to untreated controls. Exclusive exposure to AMOX/CLAV also impaired neutrophil-mediated opsonophagocytic killing, indicating reduced antibody functionality. These effects were transient, with immune parameters normalizing by 8 weeks post-prime immunization. Metabolomic and microbiome profiling revealed that maternal AMP and AMOX/CLAV differentially perturbed specific metabolite classes, including bile acids, N-acyl lipids, and indole derivatives. Key commensal taxa, including Bacteroidales and Coriobacteriales were also impacted within the gut microbiota. Together, these findings reveal a previously underappreciated maternal-offspring route of antibiotic influence that is transiently associated with neonatal vaccine responsiveness and microbiome and metabolome alterations. These results highlight maternal antibiotic exposure as a possible modifiable factor shaping early-life immunity.

## Introduction

Prophylactic administration of β-lactam antibiotics, such as AMP and penicillin, is a standard obstetric practice following prenatal identification of maternal group B *Streptococcus* (GBS) colonization. This intrapartum intervention has been robustly linked to a significant reduction in early-onset GBS disease in neonates, underscoring its critical role in contemporary perinatal infection control.^1^ ^,^ ^2^ It is estimated that up to 40% of neonates in North America are indirectly exposed to intrapartum antibiotics, regardless of delivery mode (vaginal vs. cesarean).^3^ ^,^ ^4^ Although breastfeeding supports microbial and immune development, even a single intrapartum antibiotic exposure can alter the infant gut microbiota, with changes persisting for up to three months after birth.^3^

The early postnatal period represents a critical window for immune system priming, during which gut microbial cues profoundly influence long-term immune trajectories.^5-8^ Disruption of this process, particularly from early-life antibiotic exposure, has been associated with adverse outcomes, including the emergence of disease phenotypes in adulthood (reviewed in Vangay et al.^9^), impaired vaccine responsiveness in murine models^10^ and human infants,^11-14^ increased neonatal susceptibility to sepsis^15^, and the development of antibiotic resistance. Alarmingly, global estimates indicate that healthcare professionals prescribe antibiotics to 30–50% of pregnant or lactating individuals,^16^ ^,^ ^17^ raising concerns about antibiotic resistance,^18^ disruption of neonatal microbiota, and consequent immune dysregulation.

Beyond acute effects, early antibiotic exposure has been linked, epidemiologically or experimentally, to an elevated risk of immune-mediated disorders such as atopic dermatitis, asthma, type 1 diabetes, metabolic syndrome, obesity, eosinophilic esophagitis, and inflammatory bowel disease (IBD).^19-27^ Paradoxically, under certain conditions defined by timing, dose, antibiotic class, and infant health background,^28^ ^,^ ^29^ antibiotic exposure may instead confer protection, as reported for atopic dermatitis in infants^30^ and IBD in a murine model.^30^ ^,^ ^31^ These seemingly contradictory outcomes underscore the complexity and context dependence of host–microbiome–immune system interactions and highlight the need to elucidate the immunological consequences of antibiotic-induced microbiome perturbation during this formative window.

Antibiotic exposure during infancy is consistently associated with reduced *Bifidobacterium* abundance,^32-37^ a dominant early-life taxon linked to enhanced vaccine efficacy.^11^ ^,^ ^38^ Retrospective studies further associate early antibiotic exposure with attenuated antibody responses to routine childhood immunizations,^12^ with prolonged broad-spectrum antibiotic courses causing greater suppression of vaccine-specific antibody titers than shorter narrow-spectrum regimens.^12^ To dissect the impact of antibiotic exposure on vaccine-induced immunity in offspring, we employed a murine model of early-life antibiotic exposure to examine the immunological consequences of maternal perinatal treatment with AMP, the most commonly prescribed agent in peripartum prophylaxis protocols, or the extended-spectrum combination AMOX/CLAV. We evaluated neonatal responses to the 20-valent pneumococcal conjugate vaccine (PCV20), alongside detailed gut microbiome and fecal metabolomic profiling. Our integrated analyzes uncovered distinct alterations in microbial communities and metabolic outputs that correlated with vaccine-induced humoral protection. These findings reveal a previously unknown association between the microbiome, its corresponding metabolic output, and early-life immune development.

## Materials and methods

### Ethics declarations

All animal experiments were conducted in compliance with ethical guidelines for animal research, following Institutional Animal Care and Use Committee (IACUC) regulations and approved under UC San Diego IRB protocols S00227M and S18200.

#### Murine model of antibiotic administration during gestation and postpartum

C57BL/6J (The Jackson Laboratory, JAX:000664) mice were purchased and housed throughout the experiment in filter-top cages with free access to commercial chow diet (2020X Teklad Global Soy Protein-Free Extruded Rodent Diets, Inotiv/ENVIGO) and water *ad libitum* under conditions of regulated ambient temperature (20–22 °C) and relative humidity (30–70%), with a 12 h light/12 h dark cycle. Timed pregnancies were established by pairing 8- to 10-week-old male and female mice (1:1) for 24 hours, after which females were single-housed. Pregnancy was confirmed on gestational day 14 (G14) by visible abdominal enlargement and abdominal palpation. Treatment commenced on embryonic day 16 (E16) in pregnant female mice (*n* = 18 dams; 1 mouse per cage), and mice were randomized into the designated experimental treatments, including mock (phosphate-buffered saline: PBS; Sigma Aldrich), AMP, or AMOX/CLAV. Treatment continued through day 7 (D7; Wk1) postpartum, targeting critical periods of gestation and early postpartum advancement. Mice in the antibiotic treatment arm were exposed to AMP (1 mg/mL; 150–300 mg/kg/day, assuming dams consume 4–8 ml water per day) or AMOX/CLAV (0.5 mg/mL; 75–150 mg/kg/day), which were dissolved in drinking water and provided continuously to the dams. Water was replaced every 48 hours throughout the treatment period to maintain consistent antibiotic exposure. Mice gave birth on gestation day 19 (G19), and the number of pups in each treatment group was reported. Specifically, the number of pups from PBS-, AMP-, and AMOX/CLAV-treated dams was 38 (from 6 timed-pregnant dams), 29 (from 5 timed-pregnant dams), and 45 (from 7 timed-pregnant dams), respectively. For microbiome and metabolome analyzes, fecal samples were collected from dams at week 0 (prior to initiation of oral antibiotic treatment), and subsequently at weeks 1, 2, 3, and 4 postpartum. Fecal samples from pups were collected at weeks 2, 3, and 4 post-birth. Fecal samples from pups were collected at weeks 2, 3, and 4 post-birth. For each time point, a single fecal pellet obtained from one pup was randomly selected from each independent litter (one litter per cage and dam) and included in the microbiome and metabolome analyzes, thereby minimizing the dam- and litter-associated confounding effects. Fecal pellets were carefully harvested using sterile forceps and immediately frozen at −80 °C until analysis. To assess the transfer of AMP or AMOX/CLAV to the pups, peripheral blood was collected via submandibular bleeding from both dams and pups at week 1, following the dams’ antibiotic exposure. Murine sera were isolated via centrifugation and stored at −80 °C until analysis.

Pregnant dams were randomly assigned to treatment groups. Blinding was not feasible during the animal experiments because investigators were required to know the treatment allocation to prepare and administer the assigned treatments and to maintain offspring identity according to their maternal treatment group.

#### Post-natal immunization with 20-valent pneumococcal conjugate vaccine (PCV20)

All pups were randomly assigned (per treatment) to receive intraperitoneal immunization with 100 µL of the PCV20 (AMP: *n* = 16, AMOX/CLAV: *n* = 29, Mock: *n* = 20; Pfizer), diluted 1:2 in sterile PBS, or a sham immunization with 100 µL of PBS (AMP: *n* = 13, AMOX/CLAV: *n* = 16, Mock: *n* = 18) on postnatal days 14 (D14; Wk2) and 28 (D28 or Wk4, prior to weaning). Following weaning (D28), animals were group-housed within each treatment condition, and individual pup–dam linkage was not retained for post-weaning blood collections. Peripheral blood was collected via submandibular bleeding from randomly selected PCV20-immunized mice (AMP: *n* = 6, AMOX/CLAV: *n* = 6, Mock: *n* = 6) and sham-immunized mice (AMP: *n* = 5, AMOX/CLAV: *n* = 6, Mock: *n* = 6) at weeks 2, 4, 6, and 8 following the initial immunization (prime). Murine sera were isolated by centrifugation and stored at −80 °C until analysis. Blinding was not performed during immunization because investigators were aware of treatment allocation during vaccine preparation and administration.

#### Quantification of PCV20-specific antibodies in infant murine sera

The levels of PCV20-specific antibodies, including total IgG, IgG1, IgG2b, and IgA, were quantified in serum from infant pups using enzyme-linked immunosorbent assay (ELISA). Briefly, high-binding microtiter plates were coated with 100 µL of diluted PCV20 in PBS (1:100) and incubated overnight at 4 °C. The wells were then blocked with 1% (w/v) bovine serum albumin (BSA) for 2 hours at room temperature (RT), followed by washing with PBS supplemented with 0.05% (v/v) Tween 20 (Sigma Aldrich). Serially diluted sera in PBS (100 μL) were added to the wells and incubated for 2 hours at RT. Bound antibodies were detected using HRP-conjugated goat anti-mouse IgG (BioLegend#405306, dilution: 1: 5,000), biotin-conjugated anti-mouse IgA (BioLegend#407003, dilution: 1:5,000), biotin-conjugated anti-mouse IgG1 (BioLegend#406603, dilution: 1:5,000), and biotin-conjugated anti-mouse IgG2b (BioLegend#406703, dilution: 1:5,000). Detection was performed using HRP-conjugated avidin at a 1:1,000 dilution with a TMB substrate kit (BD OptEIA). Plates were read on a plate reader at 450 nm, with background subtraction at 570 nm for correction. Serum samples were assigned coded identifiers prior to ELISA analysis to ensure investigator blinding to experimental group allocation (i.e., AMP, AMOX/CLAV, mock, sham, and PCV20). Sample identities were unblinded only after data acquisition and completion of the primary analyzes.

#### Opsonophagocytosis assay for functional antibody evaluation against *Streptococcus pneumoniae*

Opsonophagocytic killing assays were conducted using primary murine neutrophils, as described previously with modifications^39^ using *S. pneumoniae* (SPN) strain TIGR4. Mouse neutrophils were isolated from the bone marrow of naive donor mice (*n* = 2 per experiment), C57BL/6J (The Jackson Laboratory, JAX:000664), aged 6–8 weeks, and pooled prior to isolation. The MojoSort Mouse Neutrophil Isolation Kit (BioLegend) was used according to the manufacturer's instructions. An overnight culture of SPN was grown at 37 °C in Todd Hewitt broth supplemented with 2% (w/v) yeast extract (THY), and frozen glycerol stocks were prepared. The frozen stock was thawed, and bacteria were washed with 1x THY and resuspended in THY. SPN was opsonized with mouse serum for 20 minutes at 37 °C with continuous agitation. Opsonized SPN was incubated with freshly isolated mouse neutrophils (2 × 10^5^) at a multiplicity of infection (MOI) of 1: 0.01 (neutrophils:SPN) in the presence of 2% (v/v) mouse serum. Following a 1-hour incubation at 37 °C, serial dilutions were plated on blood agar plates (Hardy Diagnostics, A10BX) for enumeration of surviving colony-forming units (CFU). Opsonophagocytic killing survival rate was quantified by comparing CFU in test wells with those in control wells lacking serum but containing neutrophils, thereby establishing baseline bacterial survival in the absence of opsonophagocytic activity. Serum samples used for OPK assays were assigned coded identifiers to ensure investigator blinding to experimental group allocation (i.e., AMP, AMOX/CLAV, mock, sham, and PCV20). Colony counts were performed without knowledge of group allocation, and sample identities were unblinded only after data acquisition for downstream analysis.

#### Metabolomics

A single fecal pellet per sample was extracted with 800 μL of 50% LC-MS grade methanol and homogenized by bead beating with a 5mm bead at 25 Hz for 5 minutes, followed by a 30-minute incubation at 4 °C. The mixture was centrifuged at 15,000g for 5 minutes to pellet precipitate and solid material. The supernatant was transferred to a new 96-well plate and then vacuum-concentrated via centrifugal lyophilization (Labconco Centrivap) until ready for LC/MS analysis. The resulting supernatant was resuspended in 400 μL 50% LC/MS grade methanol in 96 well plates with 1 μL sulfadimethoxine as an internal standard. Fecal extracts were analyzed on a Thermo™ QExactive™ mass spectrometer coupled to a Vanquish Ultra-High-Performance Liquid Chromatography system (ThermoFisher). Chromatographic separation was performed on a reverse-phase HPLC C18 column (2.1 mm x 120 mm) using 0.1% formic acid in water (A) and 0.1% formic acid in acetonitrile (B) as the mobile phase. The chromatography ran in a 12-minute gradient: 0–1 min 1% B, 1–7.5 min 1–99%, 7.5–9.3 min 99%, 9.3–9.5 min 99–1%, 9.5–11 min 1% B. The column compartment was kept at 40 °C and the sample injection volume was 3 μL with a flow rate of 0.5 mL per minute. Detection was performed in electrospray ionization positive mode using a data-dependent acquisition method with the following parameters: nebulizer gas pressure at 2 bar, spray voltage was set to 3.5 kV, and sheath gas flow rate set to 54. Full MS1 scans of *m/z* 100–1300 (resolution = 35,000) were followed by MS2 acquisition of the five most intense precursor ions per scan (resolution = 17,500) at 30 eV collision energy. The raw files were converted to .mzML format using Proteowizard MZConvert software^40^ for downstream data processing and analysis. LC-MS/MS data for these fecal extractions are available in the MassIVE data repository under accession number MSV000097137. The .mzML files were analyzed in MZmine 3.9.2^41^ under the following parameters: MS1 noise factor of lowest signal 5, MS2 noise factor of lowest signal 2.5 with 1000 minimum intensity and 5000 minimum feature height with at least 4 scans per feature. The *m/z* tolerance was 0.02 or 10 ppm and the retention time tolerance was 0.1 minutes. The isotopic peaks finder, isotope grouper, and metaCorrelate modules were applied to identify multiple ion forms per molecule. The export to GNPS function exported a feature table with peak area abundances and a spectral text file. Feature-based molecular networking (FBMN) in GNPS2^42^ was applied to the filtered features with *m/z* tolerance 0.02, minimum cosine similarity score 0.7, and minimum matching peaks of 4. Library matching to “All GNPS” library and the propagated bile acid candidate library^43^ used the same tolerances. Further inspection of bile acid spectra included using previously developed MassQL queries^44^ for validation of spectra and knowledge of core structures, and manual investigation of the propagations of validated spectra within the feature based molecular network. Acylated carnitines were annotated using an in-house spectral library and manual investigation of the propagations of validated spectra within the network by a diagnostic fragment ion of 84.08 Da. The CMMC enrichment workflow in GNPS2 annotated microbial derived N-acyl lipids.^45^ Peak area data was processed in RStudio version 4.4.0 to remove features with high signal in blank extractions (sample/blank > 5) and internal standards. Internal standards were used to evaluate data quality. Prior to LC-MS/MS sample processing and data acquisition, all samples were assigned anonymized codes, and investigators were blinded to the experimental groups (i.e., AMP, AMOX/CLAV, and Mock). Group identities were revealed only after feature detection, data processing, and quality control filtering were complete. The vegan package (2.6.10) was used for rclr transformation and mixOmics (6.28.0) was used for principal component analysis (PCA) and partial least squares discriminant analysis (PLS-DA). Centroid separation in PCA was evaluated with Permutational multivariate analysis of variance (PERMANOVA). PLS-DA model performance was assessed using 4-fold cross-validation with 100 repeats. PLS-DA model performance was evaluated using leave-one-out (LOO) cross-validation and 999 permutation tests, yielding a balanced error rate (BER) of 0.42 (permutation *P* < 0.001). PLS-DA, dot plots with heatmaps, and boxplot visualizations were created using ggplot2 (3.5.1). Variable Importance Projection (VIP) scores were calculated and extracted from the PLS-DA model. Features with VIP scores > 1 that also had annotations from FBMN were retained for downstream analysis. Univariate statistical tests were performed using Wilcoxon test followed by Benjamini-Hochberg (BH) correction.

#### Microbiome analysis

The UC San Diego Microbiome Core performed nucleic acid extractions utilizing previously published protocols.^46^ Briefly, samples were purified using the MagMAX Microbiome Ultra Nucleic Acid Isolation Kit (Thermo Fisher Scientific, USA) and automated on KingFisher Flex robots (Thermo Fisher Scientific, USA). Blank controls and mock communities (Zymo Research Corporation, USA) were included per extraction plate and carried through all downstream processing steps. DNA was quantified using a PicoGreen fluorescence assay (Thermo Fisher Scientific, USA) and 16S rRNA gene amplification was performed according to the Earth Microbiome Project protocol.^47^ Briefly, Illumina primers with unique forward primer barcodes were used to amplify the V4 region of the 16S rRNA gene (515fB-806r).^48^ Amplification was performed in a miniaturized volume,^49^ with single reactions per sample.^50^ Libraries were subsequently pooled, and sequencing was carried out at the UC San Diego Institute for Genomic Medicine on the Illumina MiSeq sequencing platform with the Reagent Kit v2 and paired-end 150 bp cycles. Raw data was imported into Qiita ( Qiita #15920 )^51^ and processed using the Deblur default workflow.^52^ The resulting BIOM table was processed in QIIME 2-amplicon-2024.10.^53^ Greengenes2 was used for phylogeny and taxonomy.^54^ Samples with less than 10,000 reads were removed, and ASVs present in less than 10% of samples were removed. To control for sequencing effort, the dataset was rarefied to 10,000 reads for the Shannon diversity analysis. Feature tables were converted to .tsv format from Qiime2 into RStudio (version 4.4.0) for downstream analysis. The vegan package (2.6.10) was used for rclr transformation, and mixOmics (6.28.0) was used for PCA. Centroid separation was evaluated with PERMANOVA. Shannon entropy was calculated using the Qiime2 environment and analyzed in RStudio. Statistical significance of alpha diversity was assessed using a linear mixed-effects model for repeated measures. ALDEx2^55^ was used for differential abundance analysis of antibiotic exposure against mock exposure and BH adjusted *P* values < 0.05 were considered significant. The integrative analysis of the rclr transformed microbiome feature table and the rclr-transformed metabolomic feature table was performed using Data Integration Analysis for Biomarker discovery using Latent Components (DIABLO).^56^ To maximize covariance between groups, the DIABLO model was fit with two components and tuned to retain top features, then performance measured using a leave-one-out (LOO) cross validation with the centroids distance metric on a predefined grid of 10–20 features per block with step size = 2, with a full design matrix (off-diagonal = 1) and 500 permutations. The selected correlated features were visualized in a circos plot, where correlations >0.7 were retained.

#### Quantification and statistical analysis

Data were processed and visualized using GraphPad Prism (version 10). Results are expressed as the mean ± standard error of the mean (SEM), as indicated in the figure legends. Statistical significance (*P* < 0.05) was assessed using one-way ANOVA. Further details, including the specific statistical test used, corresponding *P*-value, sample size (number of mice), and number of biological replicates, can be found in the figure legends. Statistical analyzes for metabolomics and microbiome data are described in the respective sections.

## Results

### Peripartum exposure to AMOX/CLAV transiently attenuates PCV20-specific IgG responses and neutrophil-mediated opsonophagocytic killing in offspring

**Figure 1.**
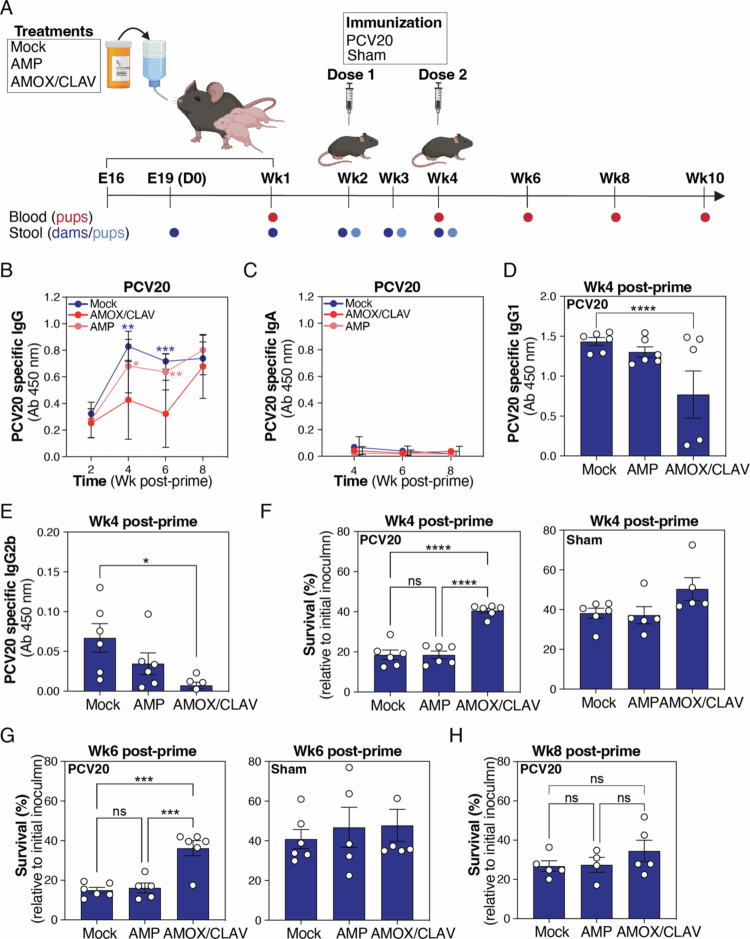
Peripartum exposure of dams to AMOX/CLAV and their immunological response to PCV20 immunization. **(A)** Schematic overview of experimental design and sampling. Dams were either left untreated (mock), administered oral AMP or oral AMOX/CLAV beginning in late prepartum (E16) and continuing through the end of first postpartum week (Wk1). Pups received either the 20-valent pneumococcal conjugate vaccine (PCV20) or a sham immunization. Blood samples (red) and stool samples (moms: dark blue; pups: light blue) were collected at the defined timepoints. Experimental details are provided in the materials and methods section. The figure was initially created with BioRender and subsequently refined in Adobe Illustrator. **(B)** Total serum anti-PCV20 IgG levels were measured following PCV20 immunization at the dose of 100 µL/mouse (PCV20 diluted 1:2 in PBS). Serum samples were collected at weeks 2, 4, 6, and 8 post-prime as described in A. The non-immunized (sham) control is shown in Figure S1A. Data are plotted as the mean ± SEM, representing 4–6 mice per group, and were analyzed by two-way ANOVA (Tukey's multiple comparisons). Wk4: Mock vs AMOX/CLAV: *P* = 0.0005, AMP vs AMOX/CLAV: *P* = 0.0355; Wk6: Mock vs AMOX/CLAV: *P* = 0.0006, AMP vs AMOX/CLAV: *P* = 0.0068. **(C)** Total serum anti-PCV20 IgA levels were measured following PCV20 immunization as described in B. Serum samples were collected at weeks 4, 6, and 8 post-prime as described in A. The non-immunized (sham) control is shown in Figure S1B. Data are plotted as the mean ± SEM, representing 5–6 mice per group, and were analyzed by two-way ANOVA (Tukey's multiple comparisons). **(D)** Total serum anti-PCV20 IgG1 levels were measured following PCV20 immunization as described in B. Serum samples were collected at week 4 post-prime. The non-immunized (sham) control is shown in Figure S1C. Data are plotted as the mean ± SEM, representing 6 mice per group, and were analyzed by one-way ANOVA (Tukey's multiple comparisons). Mock vs AMOX/CLAV: *P* = 0.0457. **(E)** Total serum anti-PCV20 IgG2b levels were measured following PCV20 immunization as described in B. Serum samples were collected at week 4 post-prime. The non-immunized (sham) control is shown in Figure S1D. Data are plotted as the mean ± SEM, representing 6 mice per group, and were analyzed by one-way ANOVA (Tukey's multiple comparisons). Mock vs AMOX/CLAV: *P* = 0.0157. **(F)** Opsonophacytic activity of anti-PCV20 IgG was evaluated using an *in vitro* opsonophagocytic killing assay. SPN was incubated (60 min) with freshly isolated murine neutrophils (MOI = 0.01) in the presence of 10% serum obtained from PCV20-immunized (left panel) or sham-immunized (right panel) mice at week 4 post-prime. Data are plotted as the mean ± SEM, representing 5–6 mice per group, and were analyzed by one-way ANOVA (Tukey's multiple comparisons). PCV20: Mock vs AMOX/CLAV: *P* < 0.0001, AMP vs AMOX/CLAV: *P* < 0.0001. **(G)** Opsonophagocytic activity of anti-PCV20 IgG was evaluated using an *in vitro* opsonophagocytic killing assay. SPN was incubated (60 min) with freshly isolated murine neutrophils (MOI = 0.01) in the presence of 10% serum obtained from PCV20-immunized (left panel) or sham-immunized (right panel) mice at week 6 post-prime. Data are plotted as the mean ± SEM, representing 5—6 mice per group, and were analyzed by one-way ANOVA (Tukey's multiple comparisons). PCV20: Mock vs AMOX/CLAV: *P* = 0.0002, AMP vs AMOX/CLAV: *P* = 0.0006. **(H)** Opsonophagocytic activity of anti-PCV20 IgG was evaluated using an *in vitro* opsonophagocytic killing assay. SPN was incubated (60 min) with freshly isolated murine neutrophils (MOI = 0.01) in the presence of 10% serum obtained from PCV20-immunized mice at week 8 post-prime. Data are plotted as the mean ± SEM, representing 4–5 mice per group, and were analyzed by one-way ANOVA (Tukey's multiple comparison).

Dams were treated with oral AMP or AMOX/CLAV from late gestation (E16) to the end of the first postpartum week (Wk1) at standard weight-based dosing (10 d total). Pups received PCV20 at Wk2 (prime) and Wk4 (boost), and PCV20-specific antibody responses were assessed as outlined in Figure 1A. Maternal AMOX/CLAV exposure significantly reduced total PCV20-specific IgG titers at 4 and 6 weeks post-prime compared with pups from AMP-treated or untreated dams, an effect that was resolved by week 8 post-prime (Figure 1B). Subclass analysis demonstrated significantly lower IgG1 and IgG2b titers in AMOX/CLAV-exposed pups at week 4 (Figure 1D,E), whereas PCV20-specific IgA remained uniformly low across groups (Figure 1C).

Functional antibody activity, measured by neutrophil opsonophagocytic killing (OPK), was likewise reduced in AMOX/CLAV-exposed pups at weeks 4 and 6 post-prime (Figure 1F,G) and recovered by week 8 (Figure 1H).

### Maternal antibiotic exposure affects gut microbiota composition in dams and offspring

**Figure 2.**
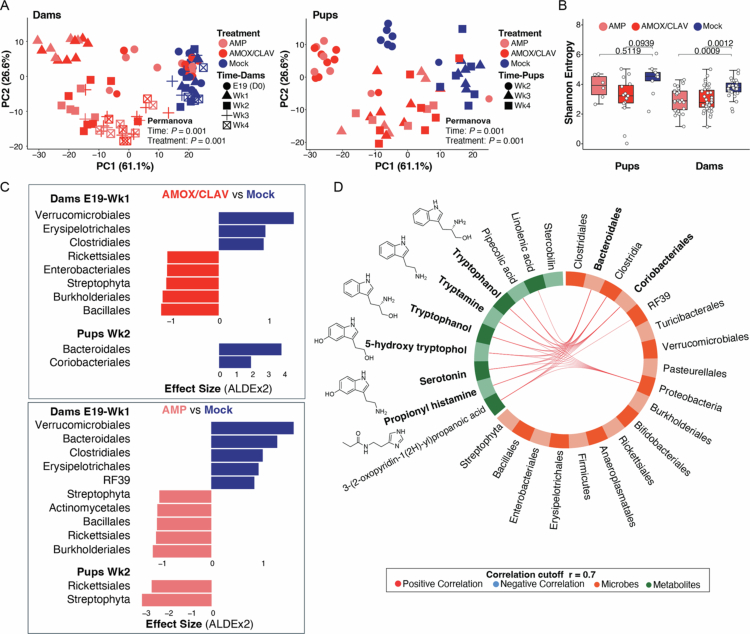
Mice exposed to antibiotics show altered microbiomes. **(A)** Principal component analysis (PCA) of rclr-transformed microbial features revealed overall separation of microbial communities between mice exposed to antibiotic treatment or mock treatment. Separation was assessed using PERMANOVA, showing a significant effect of both treatment condition and time in pups and dams that were indirectly or directly exposed to antibiotics, respectively (PERMANOVA: treatment *P* = 0.001, time *P* = 0.001). **(B)** Shannon diversity was significantly reduced in dams receiving antibiotic treatments (*P* < 0.01, linear mixed-effects model (LME) with subject as random effect). Similarly, pre- and post-immunized PCV20 Wk2–4 pups that were exposed indirectly to AMOX/CLAV showed depleted Shannon diversity (*P* = 0.09, linear mixed-effects model for repeated measurements) compared to no observed difference between AMP and Mock (LME: *P* = 0.51). The boxplots represent the first (lower) and third (upper) quartiles, with the boxes indicating the interquartile range (IQR). **(C)** Microbial orders driving differences between antibiotic treatment conditions at early time points (Dams E19-Wk1, Pups Wk2) were identified using differential abundance analysis with ALDEx2 (FDR-corrected *P* < 0.05). **(D)** Multi-omics integration of the top 10 rclr-transformed annotated microbial features, collapsed at the order level, and top 10 rclr-transformed annotated metabolic features revealed strong correlations, visualized using a Circos plot generated with DIABLO. Corrections greater than 0.7 were retained. Negative (none detected) and positive correlations are depicted in blue and red, respectively.

PCA of robust center log-ratio (rclr)-transformed data revealed distinct clustering by treatment and time in both dams and pups (Figure 2A), with antibiotic exposure and sampling time exerting strong effects on fecal microbiome composition (PERMANOVA; dams: treatment: *P* = 0.001, R^2^ = 0.37634, F = 81.2695, time: *P* = 0.001, R^2^ = 0.31288, F = 33.783; pups: treatment: *P* = 0.001, R^2^ = 0.27040, F = 25.0924, time: *P* = 0.001 R^2^ = 0.46395, F = 43.0531). No clear separation was observed between the AMP and AMOX/CLAV groups. In dams, samples collected during active treatment (E19 and Wk1) clustered separately from post-treatment samples (Wk2–4), indicating a shift between exposure and recovery phases. In pups, Wk2 microbiomes clustered distinctly from those collected at Wk3–Wk4, reflecting temporal maturation following maternal treatment. Alpha diversity was reduced in antibiotic-treated dams compared with controls (*P* < 0.01; Figure 2B). In pups, Shannon diversity was similar across groups (Figure S2A). Among immunization samples, AMOX/CLAV-exposed pups showed a non-significant trend toward reduced diversity compared with controls (*P* < 0.1), whereas AMP exposure did not impact diversity (*P* = 0.51) (Figure 2B). These findings suggest a potential trend toward reduced gut microbial richness in pups that exhibited reduced vaccine responses.

To further explore the taxonomic drivers of these differences, we performed differential abundance analysis using ALDEx2 (Figure 2C). Samples were stratified by the time point and vaccination status identified in earlier analyzes (Figure 2A,B), and features annotated against the Greengenes phylogeny were collapsed at the order level.

During active antibiotic treatment, AMOX/CLAV-treated dams exhibited significant depletion of several commensal orders, including Verrucomicrobiales, Erysipelotrichales, and Clostridiales, with concurrent enrichment of Bacillales and Burkholderiales (Figure 2C) (*P* < 0.05). AMP-treated dams similarly showed depletion of Verrucomicrobiales, Bacteroidales, and Clostridiales, alongside enrichment of Burkholderiales, Rickettsiales, and Bacillales. Notably, post-treatment samples contained a greater number of significantly enriched or depleted taxa than samples collected during active exposure (AMP: 19 vs. 6; AMOX/CLAV: 19 vs. 4). Although many changes overlapped between treatment groups, post-AMOX/CLAV treatment uniquely showed depletion of Lactobacillales and enrichment of Pseudomonadales and Pasteurallales (Figure S3A), whereas post-AMP treatment uniquely showed depletion of Erysipelotrichales.

At Wk2 (prior to PCV20 immunization), pups from AMOX/CLAV-treated dams exhibited selective depletion of Bacteroidales and Coriobacteriales compared with mock controls, whereas no significantly depleted taxa were observed in AMP-exposed pups (Figure 2C). In contrast, AMP-exposed pups showed enrichment of Streptophyta and Rickettsiales. The similarity between dam and offspring community profiles indicates maternal transmission of antibiotic-induced microbiome perturbations. By Wk3–4, AMP-exposed, PCV20-immunized pups exhibited enrichment of Turicibacterales and Clostridiales (Figure S3A, right panel). AMOX/CLAV-exposed, PCV20-immunized pups remained uniquely depleted for Bacteroidales and Coriobacteriales, while showing enrichment of Clostridiales, Rickettsiales, Bacillales, Bifidobacteriales, Streptophyta, and Burkholderiales, (Figure S3A, left panel). These patterns indicate that AMOX/CLAV drives more sustained depletion of commensal anaerobes associated with early immune education.

### Maternal antibiotic exposure induces coordinated and pathway-specific metabolomic shifts in offspring

Untargeted metabolomics of fecal samples was performed alongside microbiome profiling and integrated using DIABLO, a supervised multi-omics framework designed to identify features that covary across datasets. Feature selection was guided by optimization of a partial least squares (PLS) model, and metabolite-taxon associations with correlation coefficients >0.70 were retained for further prioritization. The DIABLO model achieved centroid distance error rates of 0.0625 (component 1) and 0.000 (component 2) for the metabolome data, and 0.1250 (component 1) and 0.1875 (component 2) for the microbiome data, indicating strong discriminatory performance across both data blocks. This approach revealed strong associations between the relative abundances of Bacteroidales and Coriobacteriales and eight metabolites of interest, six of which possessed tryptophan-derived indolic structures, a class of metabolites known to mediate host–microbe and host–immune interactions.^57-59^ These included putative tryptophanol, tryptamine, 5-hydroxytryptophol, serotonin, and propionyl-histamine (Figure 2D). Although the order-level taxonomic resolution limits direct attribution of metabolite production to specific bacterial genera or strains, this coordinated depletion of these metabolites in AMOX/CLAV-exposed pups likely reflects disruption of a broader microbial metabolic axis with known immunomodulatory roles, rather than taxon-specific biosynthetic activity.

**Figure 3.**
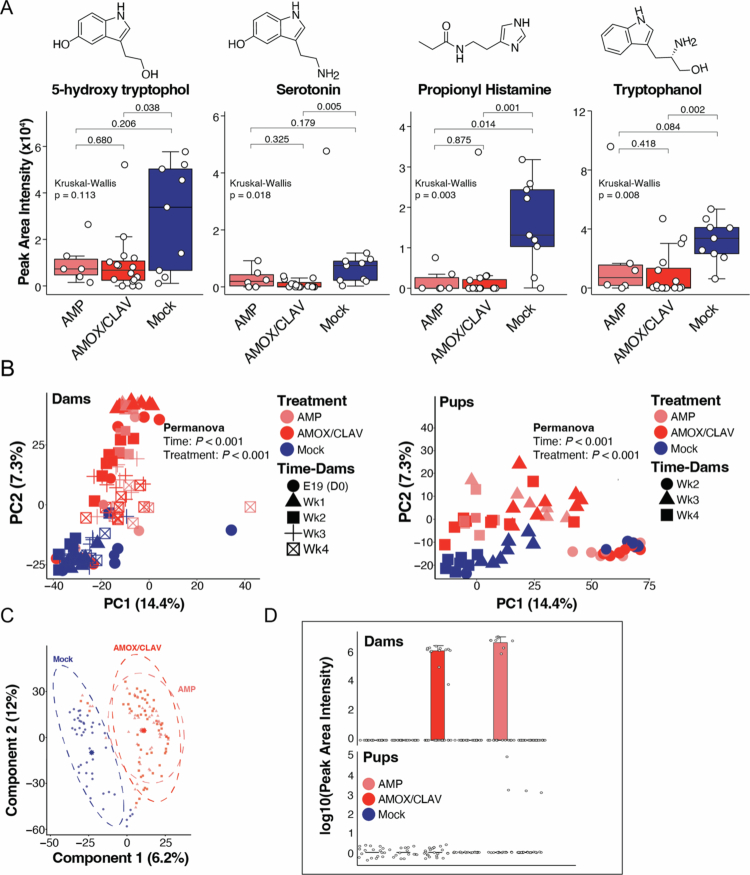
Metabolomics analysis of mice stratified by treatment condition. **(A)** Univariate analysis of selected molecular features 5-hydroxy tryptophol (feature ID 808), serotonin (feature ID 805), propionyl histamine (feature ID 481), and tryptophanol (feature ID 2566). Levels of 5-hydroxy tryptophanol, serotonin, tryptophanol, and propionyl histamine in pre- and post-immunized PCV20 Wk2–Wk4 pups that were indirectly exposed to antibiotics. Statistical significance was assessed using the *Kruskal-Wallis test followed by Dunn’s test.* The boxplots represent the first (lower) and third (upper) quartiles, with the boxes indicating the interquartile range (IQR). **(B)** PCA of rclr-transformed metabolomic features revealed distinct clustering of mice based on antibiotic exposure. Separation was assessed using PERMANOVA, showing a significant effect of both treatment condition and time in pups and dams that were indirectly or directly exposed to antibiotics, respectively (PERMANOVA: treatment *P* < 0.001, time *P* < 0.001). **(C)** Partial Least Squares Discriminant Analysis (PLS-DA) of RCLR-transformed metabolomics features stratified by antibiotic treatment, with 95% confidence ellipses. Model performance was evaluated by cross-validation yielded a balanced error rate of (BER) ≈ 0.43, indicating moderate classification accuracy. **(D)** Univariate analysis of detected features annotated as AMP or AMOX/CLAV presented as boxplots of summed log_10_(Peak area + 1)-transformed intensities in all treatment groups congregated at all time points. AMP and AMOX/CLAV were detected in the dams of their respective treatment groups but were not detected in the pups' feces.

Univariate testing showed significant reductions in peak area abundance of 5-hydroxy tryptophol, serotonin, and tryptophanol in AMOX/CLAV-exposed pups relative to mock-exposed PCV20-immunized controls (Wilcoxon, FDR-corrected *P* < 0.05) (Figure 3A), mirroring the attenuated IgG vaccine responses observed in the same cohort (Figure 1B). Propionyl-histamine levels were reduced in both antibiotic-exposed groups, with a greater reduction observed in the AMOX/CLAV cohort (Figure 3A). Importantly, this indole depletion phenotype was not dependent on vaccination, as analysis restricted to PBS-immunized pups showed similar reductions in these indolic-derived metabolites following antibiotic exposure (Figure S4A). Thus, the metabolite shifts represent antibiotic-induced microbiome perturbation, rather than vaccine-driven metabolic changes.

### Global metabolomic signatures distinguish antibiotic-exposed and control groups

**Figure 4.**
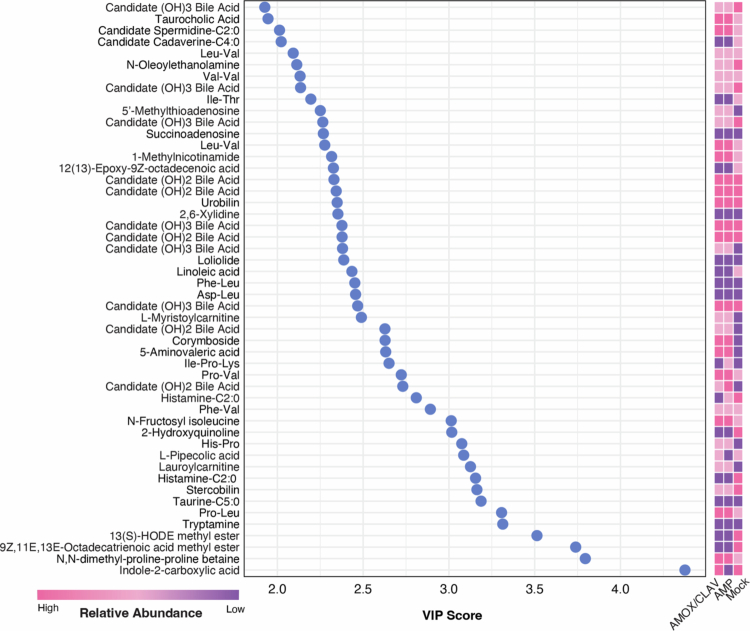
Heatmap and dot plot associated with the metabolomics analysis of mice stratified by treatment condition. The dot plot and heatmap highlight the top 50 annotated metabolites ranked by VIP scores. The heatmap represents the relative abundance of each feature detected across the treatment groups. The annotations are based on MS/MS matching and correspond to MSI annotation Levels 2 or 3.^60^ This means that the metabolite identities have not been confirmed using authentic standards. Therefore, the identification of a specific metabolite name does not exclude the possibility of other structural isomers.

Unsupervised PCA revealed a clear separation between antibiotic-treated and mock-treated dams and offspring, driven by both treatment and sampling time (PERMANOVA; dams: treatment: *P* = 0.001, R^2^ = 0.46233, F = 56.3932, time: *P* = 0.001, R^2^ = 0.12990, F = 7.9223; pups: treatment: *P* = 0.001, R^2^ = 0.11880, F = 9.2615, time: *P* = 0.001 R^2^ = 0.55423, F = 43.2055; Figure 3B). To interrogate treatment-specific metabolic signatures, we next applied a supervised PLS-DA model across all samples, comparing AMP-, AMOX/CLAV-, and mock-treated groups. This analysis again distinguished both antibiotic-treated cohorts from mock controls (Figure 3C). Key discriminating metabolites were identified using Variable Importance in Projection (VIP) scores from component 1, retaining annotated metabolites with VIP > 1. The top 50 VIP-ranked metabolites were visualized in a VIP dot plot and corresponding abundance heatmap, revealing that both antibiotic groups exhibited similar overall intensity patterns that were distinct from those of the mock group (Figure 4). These features included putative bile acids, N-acyl lipids, dipeptides, and an acyl carnitine, among other small molecules. Notably, although AMP and AMOX/CLAV were detectable in dams during the treatment window (Figure 3D, upper panel), neither antibiotic was detected in pup feces (Figure 3D, lower panel) or serum (Table S1).

### Perturbation of microbial bile acid and N-acyl lipid metabolism in dams and offspring

Bile acid metabolism was examined by categorizing annotated features into three classes: non-conjugated bile acids, taurine-conjugated bile acids, and bile acids conjugated to alternative amines (e.g., leucine, phenylalanine, and cysteamine). Antibiotic treatment elicited distinct and time-dependent shifts in bile acid composition in both dams and pups (Figure S5A,B). At E19 and Wk1, both antibiotic-treated dam groups showed reduced levels of non-conjugated (free) bile acids and amine-conjugated bile acids (excluding taurine- and glycine-conjugated) relative to mock controls (Figure S5A,B). In AMOX/CLAV-treated dams, the abundance of non-conjugated bile acids increased significantly from E19/Wk1 to Wk2–4 (*P* < 0.0001), accompanied by a significant rise in amine-conjugated bile acids (*P* < 0.01) (Figure S5A). AMP-treated dams showed a similar directional trend, though the changes did not reach statistical significance (Figure S5A). In contrast, taurine-conjugated bile acids declined over time in both AMP (*P* < 0.01) and AMOX/CLAV (*P* < 0.001) groups (Figure S5A); whereas mock-treated dams exhibited stable bile acid profiles across time (Figure S5A). By Wk4, three weeks after cessation of antibiotic treatment, bile acid profiles converged across all dam groups (Figure S5B). In antibiotic-treated pups, Wk2 samples showed a pronounced reduction in all bile acid classes relative to controls, with gradual recovery over time, trending toward mock-like levels by Wk4 (Figure S5B).

Beyond bile acid metabolism, antibiotic exposure also affected broader lipid pathways. Acylcarnitine levels were elevated in both dams and pups exposed to antibiotics (Figure S6A). In contrast, microbially derived N-acyl lipids were reduced during antibiotic exposure, with partial recovery by Wk2 (Figure S7A). Together, these data indicate that peripartum antibiotic exposure is associated with alterations in microbiota-controlled bile acid and lipid signaling pathways that areknown to support early immune maturation.

## Discussion

Maternal antibiotic exposure during late gestation and lactation, particularly to broad- or extended-spectrum agents, perturbs early immune maturation and delays the establishment of effective vaccine-induced protection. Early-life immunity is tightly regulated by host-microbiota interactions, especially during the perinatal period when initial microbial colonization shapes developing immune networks and vaccine responsiveness.^61^ Immunostimulatory microbial metabolites have been proposed to function as endogenous signals that engage pattern recognition receptors on innate immune cells, potentially influencing vaccine efficacy across diverse modalities.^61^ ^,^ ^62^ Using a murine model, we examined the consequences of maternal antibiotic exposure on neonatal responses to PCV20. We found that maternal treatment with AMOX/CLAV, but not AMP, impaired PCV20-induced antibody responses and neutrophil-mediated OPK activity in offspring. Although these effects were transient, the disruption occurred during a critical window of neonatal susceptibility, when delayed or suboptimal immune protection may increase infection risk.^63^ These findings are consistent with previous human^12^ and murine^10^ studies demonstrating that maternal antibiotic exposure can attenuate infant vaccine responses, particularly following broad-spectrum or high-dose antibiotic regimens. It is notable that the reduction in OPK activity was transient, with recovery observed by 8 weeks in our *in vitro* system. These findings support a model of short-lived immune modulation rather than sustained impairment. Further studies, particularly *in vivo*, over longer timeframes, and incorporating pathogen challenge models, will be important to determine whether such transient changes have functional consequences for immune development and host defense during early life.

The mechanistic basis for the loss of vaccine protection is likely multifactorial. Disruption of the maternal gut microbiota alters the pool of microorganisms available for vertical transmission at birth and during lactation, impairing neonatal gut colonization and the microbial signaling required for immune priming.^5-9^ Additionally, antibiotic-induced modulation of placental or mammary immunological signaling could contribute to altered fetal and neonatal immune programming.^15^ ^,^ ^64^ ^,^ ^65^

We further explored how maternal antibiotic exposure influences immune development through host-microbe pathways. Prior work has shown that perinatal antibiotics alter both maternal microbiome composition and metabolite profiles, with downstream effects on infant health.^66^ Consistent with these observations, maternal antibiotic treatment in our study led to coordinated shifts in the maternal gut microbiota and metabolome, with corresponding alterations observed in the offspring, supporting maternal-to-infant transmission of microbial community states. 16S rRNA sequencing revealed selective enrichment of Bacteroidales and Coriobacteriales in AMOX/CLAV-exposed pups—taxa previously implicated in regulating inflammatory tone,^67^ T cell activation,^68^ and adaptive immune responses.^69^ Notably, this taxonomic disruption coincided with reduced PCV20-specific antibody responses, suggesting that the loss of these lineages may impair immune priming.

We assessed the detectability of AMP and AMOX/CLAV in dam and pup samples to evaluate systemic exposure. The absence of detectable levels in pup serum and fecal matrices suggests limited direct exposure or concentrations below the assay detection threshold. These findings are consistent with the possibility that early-life immune development is influenced indirectly through maternally shaped microbial and metabolic environments. Although detection limits for AMOX/CLAV and AMP are inherently dependent on both analytical method and sample matrix, and targeted quantification was not performed in this study, prior LC–MS/MS analyzes of human plasma using comparable extraction and acquisition strategies have reported lower limits of quantification for amoxicillin down to 10 ng/mL.^70^ Moreover, recent human pharmacokinetic studies have demonstrated that amoxicillin is transferred into human breast milk at low concentrations, with an estimated relative infant dose of <0.4%, indicating minimal infant exposure during maternal therapy.^71^ Given these considerations, the absence of detectable antibiotic signal in both serum and fecal samples from pups supports the interpretation that the observed microbiome alterations are unlikely to reflect direct antibiotic exposure, but rather arise indirectly through maternally mediated microbial and metabolic changes.

Both AMP and AMOX/CLAV exposure were associated with overlapping metabolic alterations, including elevated host-derived taurocholic acid^72^ and acylated carnitines,^73^ alongside depletion of microbiota-derived N-acyl lipids, which are known to support T cell differentiation and mucosal immune function^45^. These results reflect previous studies that have found elevated levels of the same carnitines, including butyryl carnitine, decanoyl carnitine, dodecenoyl carnitine, and host-derived bile acids in perinatal exposure to AMP.^65^ However, AMOX/CLAV exposure was associated with additional, distinct changes, characterized by depletion of non-conjugated bile acids,^72^ amine-conjugated bile acids, and tryptophan-derived indole metabolites.

These metabolite classes signal through bile acid receptors and the aryl hydrocarbon receptor (AhR) to maintain epithelial integrity, regulate immune tone, and calibrate mucosal adaptive responses.^74-76^ Thus, AMOX/CLAV exposure is selectively associated with a microbiota-dependent metabolic signaling network involving bile acids, N-acyl lipids, and indole-AhR ligands—pathways that are collectively associated with effective vaccine-induced immunity.

For example, taurocholic acid, which was elevated in both antibiotic treatment groups, has been shown to promote the expansion of pathogenic bacteria *in vivo*.^77^ Antibiotic exposure also resulted in depletion of bile amidates, microbial products implicated in gut–immune communication,^78^ ^,^ ^79^ and accompanied reduced levels of microbially derived (non-conjugated) bile acids and elevated levels of host-conjugated bile acids.^80^ In parallel, N-acyl lipids, which were depleted in both AMP- and AMP/CLAV-treated groups, are produced by >70 commensal species and contribute to T cell differentiation and mucosal immune function.^45^ The observed depletion of short-chain fatty acid (SCFA)-derived N-acyl lipids likely reflects diminished SCFA biosynthetic capacity, although SCFAs themselves were not directly annotated in this study. N-acyl lipids contribute to immune cell differentiation and inflammatory regulation, whereas SCFAs are well established as key mediators of immune homeostasis and vaccine responsiveness.^81^

Integrative multi-omics analysis revealed strong correlations between immune-associated microbial taxa and tryptophan-derived indole metabolites. However, the limited taxonomic resolution and absence of functional knockout models preclude causal interpretation, and these findings should therefore be interpreted as associative rather than mechanistic. Several such metabolites, including indolelactic acid (ILA), indolepropionic acid (IPA), and indoleacetic acid (IAA), are produced by commensal bacteria in the infant gut.^82^ Signaling through AhR, these indole derivatives can influence mucosal barrier integrity, T follicular helper (Tfh) cell differentiation, germinal center formation, and cytokine signaling.^83-85^ Additional tryptophan-derived metabolites detected here—including serotonin, tryptamine, tryptophol, and 5-hydroxy tryptophol, as well as two isobaric tryptophanol species—were significantly depleted in AMOX/CLAV-exposed infants. Members of the genera *Bifidobacterium* and *Bacteroides* spp. are recognized producers of these metabolites;^86^ however, given that our analysis is resolved at the order level, functional inferences remain limited to broad taxonomic groups and do not permit strain-level or genus-specific attribution. Taken together, these findings suggest that the disruption of Bacteroidales-mediated tryptophan metabolism may reduce the availability of AhR-active microbial metabolites within the host, and is associated with impaired mucosal immune signaling and suboptimal germinal center responses, ultimately coinciding with reduced vaccine-specific antibody function.

Exposure to unnecessary broad-spectrum antibiotics remains widespread.^87^ In fact, broad spectrum AMOX/CLAV is currently evaluated for use as an orally administered prophylactic agent for reducing GBS and other infection-related risks.^88-91^ AMOX/CLV is associated with a higher risk of neonatal necrotizing enterocolitis in cases of preterm pre-labor rupture of membranes,^85^ ^,^ ^92^ which has historically led to strong clinical advice against its prophylactic use. Our finding, if further corroborated in human studies, could lead to stronger contraindication for its use in pregnant women near term.

The absence of detectable antibiotics in pup serum and feces suggests that direct pharmacologic exposure in the offspring is unlikely or below the detection threshold. This supports an indirect mechanism in which maternal microbiome perturbation and/or alterations in breast milk composition may contribute to the observed microbial and immune phenotypes.

In our study, the maternal microbiome was perturbed through AMP or AMOX/CLAV administered orally in drinking water to the dams, rather than via the intravenous (IV) route recommended in clinical guidelines. This approach was used to avoid infant cannibalization associated with distress in dams subjected to repeated IV procedures. Oral administration of antibiotics could lead to perturbations of the maternal microbiome that differ from those induced by the IV route. However, to our knowledge, no studies directly compare AMOX/CLAV administered in drinking water versus the IV route. Therefore, a precise conclusion regarding how the selected route of antibiotic administration impacted our results cannot be drawn. Nevertheless, available pharmacokinetic (PK) data provide some insight.

We did not identify studies comparing levels of AMOX/CLAV in breast milk following oral versus IV administration in mice or humans. However, in humans, orally administered AMOX/CLAV is approximately 80–93% bioavailable.^93^ Thus, we presume that oral versus IV administration of AMP or AMOX/CLAV may result in broadly similar systemic exposure, although this does not necessarily translate to equivalent effects on the breast milk microbiome.

In the gut, orally administered AMOX/CLAV delivers an estimated 7–23% of the dose for amoxicillin ^93^ ^,^ ^94^, and likely higher for clavulanic acid, whereas IV administration results in gut exposure primarily through the relatively minor biliary secretion pathway ^95^. Nonetheless, IV AMOX/CLAV has been shown to cause significant gut microbiome disruption. For example, the ARMORD study reported that IV co-amoxiclav was associated with substantial reductions in microbiome diversity and anaerobe abundance, comparable in magnitude to piperacillin–tazobactam and meropenem.^96^

We demonstrate that AMOX/CLAV selectively perturbs this microbial–metabolic axis, whereas AMP does not, thereby allowing molecular investigation of the specific pathways underlying sub-optimal neonatal vaccine responsiveness observed *in vivo* (Figure S8). Although the study is preclinical, these findings suggest that minimizing unnecessary exposure to AMOX/CLAV during pregnancy and lactation may help to preserve early-life vaccine responsiveness and immune function. However, these observations are derived from a preclinical model in which antibiotics were administered orally, and thus require validation in humans to establish their translational relevance. It remains to be determined whether early life vaccine responsiveness and immune function would be similarly affected under alternative routes of administration, including intravenous delivery in clinical settings. Future studies should further dissect strain-level contributions within Bacteroidales and Coriobacteriales, define the immune cell subsets most sensitive to perturbation (e.g., Tfh, Treg, and germinal center B cells), and evaluate whether targeted microbial or metabolite-based interventions can restore vaccine responsiveness during the neonatal immune development window.

## Limitations

This study examined the metabolic mechanisms underlying microbially mediated immunity in early life, leveraging high-throughput sequencing and high-resolution mass spectrometry for metabolite identification. Several limitations merit consideration. This multi-omics analysis leverages coordinated profiling of microbial metabolites and host immune responses to identify candidate associations but does not establish causality. Experimental validation, including metabolite supplementation or rescue approaches, will be required to determine whether these metabolites directly modulate vaccine-induced immunity. Metabolites were annotated by MS/MS spectral matching (Metabolomics Standard Initiative level 2),^60^ against GNPS spectral libraries. Although AMP and AMOX/CLAV were detected in the feces of antibiotic treated dams, they were not detected in pup feces or serum. This absence may reflect limited transfer of antibiotics to offspring and/or analytical sensitivity constraints. Future studies should incorporate higher-sensitivity instrumentation and targeted quantification approaches to determine whether these antibiotics are transmitted through breast milk. While oral AMP or AMOX/CLAV dosing effectively perturbed the maternal gut microbiota, it does not fully mirror the intravenous regimens commonly used in clinical intrapartum antibiotic prophylaxis; consequently, systemic exposure and transfer to offspring may differ. Nevertheless, this model was suitable for interrogating microbiome-mediated effects on early immune development while maintaining offspring viability, as minimizing maternal stress reduces the risk of cannibalism and pup loss, an important consideration in neonatal studies. Additionally, although the sample size was limited, the pronounced effects observed in this study replicate or align with multiple prior studies of antibiotic-induced gut microbiome perturbations, thereby strengthening confidence in our conclusions. Finally, post-weaning serum analyzes could not be linked to litter of origin due to redistribution of animals at weaning, precluding explicit modeling of intra-litter dependence. Although pups were sampled across multiple dams within each group and selection was randomized, residual litter effects may still contribute to variability in antibody responses and should be considered in the interpretation.

## Supplementary Material

R2_Supplementary File_Suzuki et al_07142026.docx R2_Supplementary File_Suzuki et al_07142026.docx

Supplementary Material BioRender_Publication License 23062026_Figure S8.pdf

## Data Availability

All data required to evaluate the paper’s conclusions are included in the manuscript and/or Supporting Information. Metabolomics and microbiome data have been deposited in GNPS/MassIVE as well as Qiita with a dataset identifier MSV000097137 and 15920, respectively. The EBI accession number is ERP186116. The source code, statistical analyzes and datasets are available under the assigned DOIs 10.5281/zenodo.17726093 and 10.5281/zenodo.17728254.
