# Supplementary material for: Effect of perinatal ampicillin or amoxicillin/clavulanate exposure on maternal and infant gut microbiome, metabolome, and infant responses to the 20-valent pneumococcal conjugate vaccine
Source: Gut Microbes Rep. 2026 Jul 20;3(1):2705707. doi: 10.1080/29933935.2026.2705707 (PMC13387116; doi:10.1080/29933935.2026.2705707)
Supplement: R2_Supplementary File_Suzuki et al_07142026.docx [file KGMR_A_2705707_SM1601.docx]

**Supplemental Information for**

Effect of Perinatal Ampicillin or Amoxicillin/Clavulanate Exposure on Maternal and Infant Gut Microbiome, Metabolome, and Infant Responses to the 20-valent Pneumococcal Conjugate Vaccine

Emi Suzuki^a,b,c,1^; Victoria Deleray^d,1^; Jasmine Zemlin^d,1^; Armin Kousha^a^; Hannah Nonoguchi^a^; Daniel Sun^a^; Chih-Ming Tsai^a^; Simone Zuffa^d^; Kine Eide Kvitne^d^; Pieter C. Dorrestein^d^; Shirley M. Tsunoda^d^; Victor Nizet^a,d^*;* George Y. Liu^a,b,2^*^*^;* Fatemeh Askarian^a,2*^

^a^Department of Pediatrics, University of California San Diego, La Jolla, CA, USA. ^b^Division of Infectious Diseases, Rady Children’s Hospital, San Diego, CA, USA. ^c^Division of Gastroenterology, Hepatology, and Nutrition, Rady Children’s Hospital, San Diego, CA, USA; ^d^Skaggs School of Pharmacy and Pharmaceutical Sciences, UC San Diego, La Jolla, CA, USA.

^1^Equally contributed.

^2^Equally contributing senior authors.

^*^To whom correspondence should be addressed:

**E-mail:** [faaskarian@health.ucsd.edu](mailto:faaskarian@health.ucsd.edu); [gyliu@health.ucsd.edu](mailto:gyliu@health.ucsd.edu)

**Tel:** (+1) 858-534-2325; (+1) 858-246-5830

**Keywords**

Perinatal antibiotic exposure, vaccine responsiveness, microbiome-metabolome interactions

**This file includes:**

Supplementary Figures (including figure legends) S1–S8

Supplementary Table (Table S1)

**
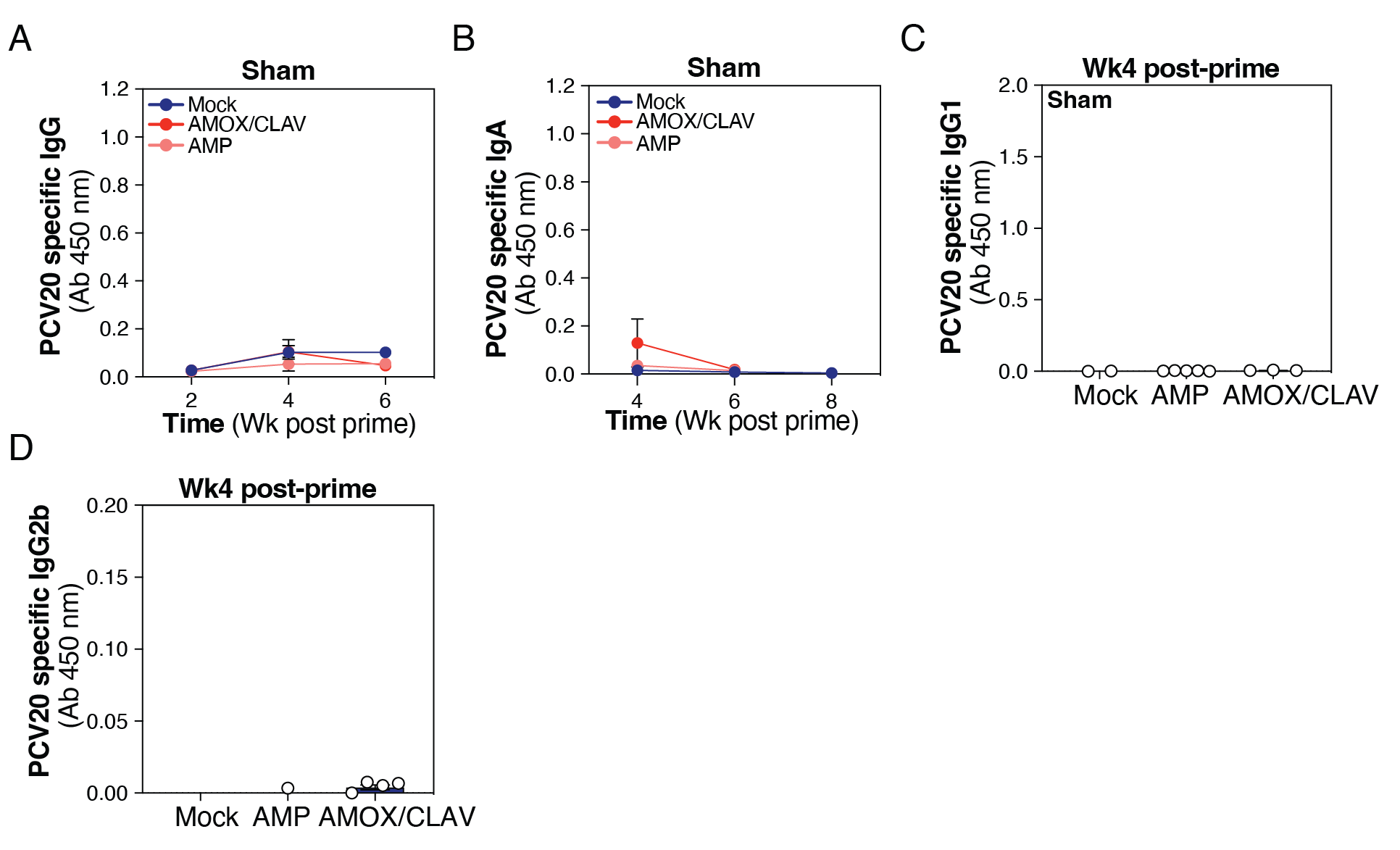
**

**Figure S1. Peripartum exposure of dams to AMOX/CLAV and their immunological response to PCV20 immunization. (A)** Total serum anti-PCV20 IgG levels were measured following sham immunization (PBS) at the dose of 100 µL/mouse. Serum samples were collected at weeks 2, 4, and 6 post-prime as described in Fig. 1A. The PCV20-immunized mice are shown in Fig. 1B. Data are plotted as the mean ± SEM, representing 5–6 mice per group. **(B)** Total serum anti-PCV20 IgA levels were measured following sham (PBS) immunization as described in A. Serum samples were collected at weeks 4, 6, and 8 (mock) post-prime. The PCV20-immunized mice are shown in Fig. 1C. Data are plotted as the mean ± SEM, representing 5–6 mice per group. **(C)** Total serum anti-PCV20 IgG1 levels were measured following sham (PBS) immunization as described in A. Serum samples were collected at week 4 post-prime. The PCV20-immunized mice are shown in Fig. 1D. Data are plotted as the mean ± SEM, representing 5–6 mice per group. **(D)** Total serum anti-PCV20 IgG2b levels were measured following sham (PBS) immunization as described in A. Serum samples were collected at week 4 post-prime. The PCV20-immunized mice are shown in Fig. 1E. Data are plotted as the mean ± SEM, representing 5–6 mice per group.


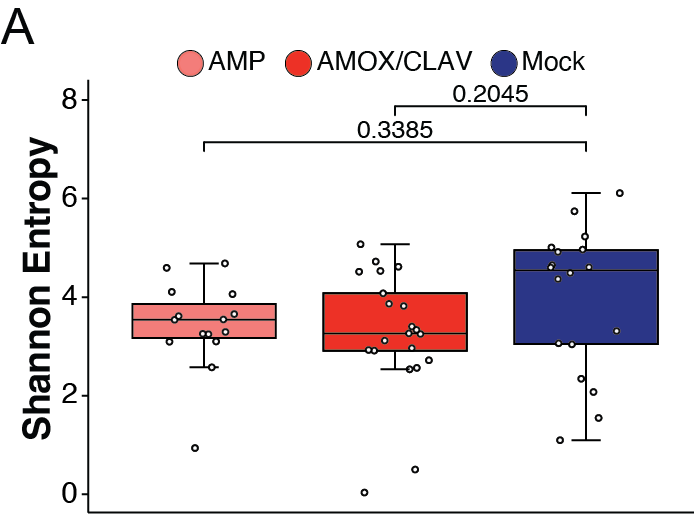


**Figure S2**. **Alpha diversity analysis of Pups Wk2-Wk4.** **(A)** Shannon diversity was not significantly reduced (*P* < 0.05) in pups, regardless of their immunization status, exposed to antibiotic treatments. Statistical significance was assessed using a linear mixed-effects model for repeated measures. The boxplots represent first (lower), interquartile range (IQR), and third (upper) quartile.


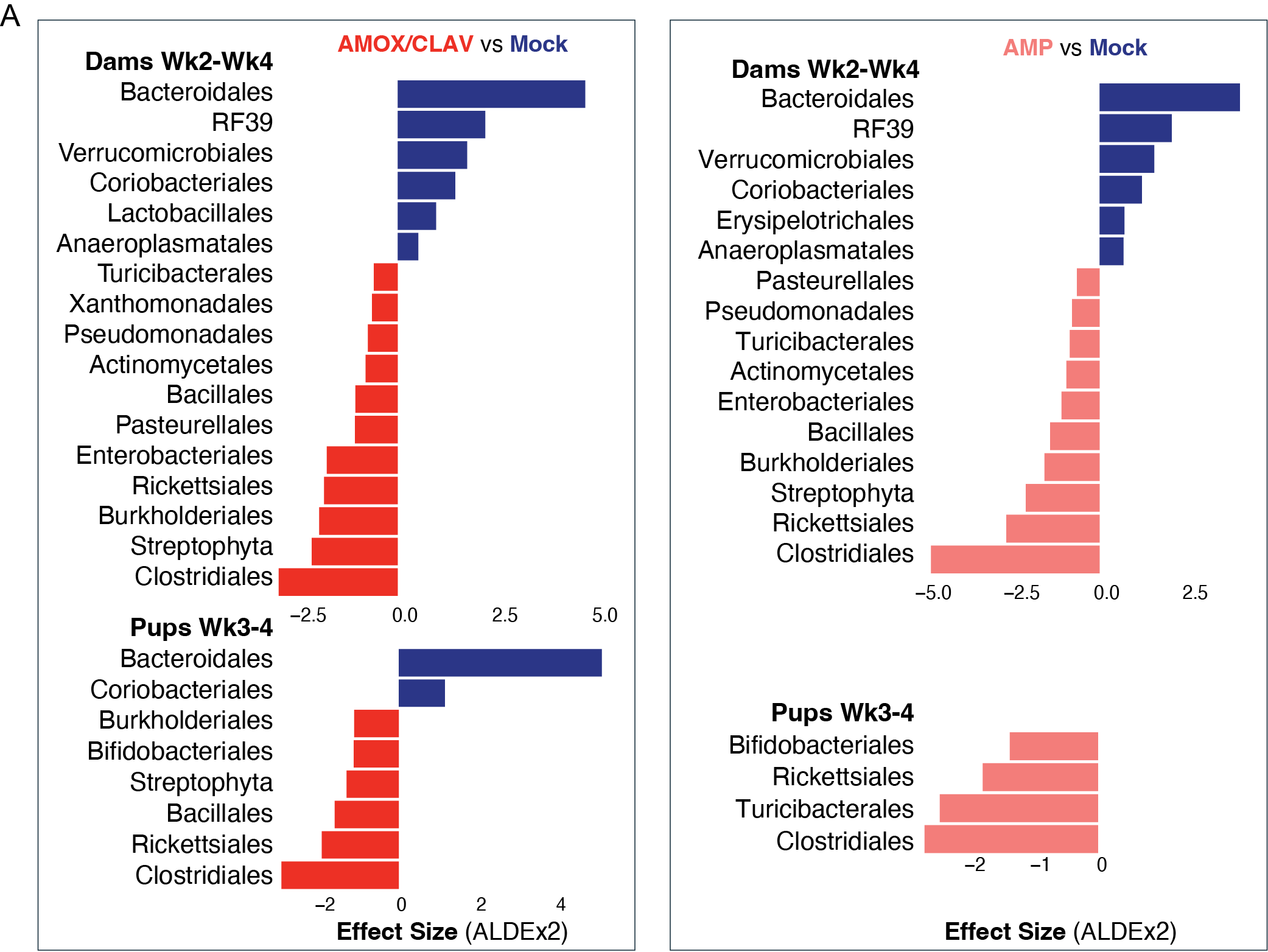


**Figure S3. Microbiome analysis of mice stratified by treatment condition** **(A)** Key microbial features driving differences between antibiotic treatment conditions at later time points (dams, Wk2-Wk4; PCV20-immunized pups, Wk3-Wk4), collapsed at the order level, were identified using differential abundance analysis with ALDEx2 (FDR-corrected *P* < 0.05).

**
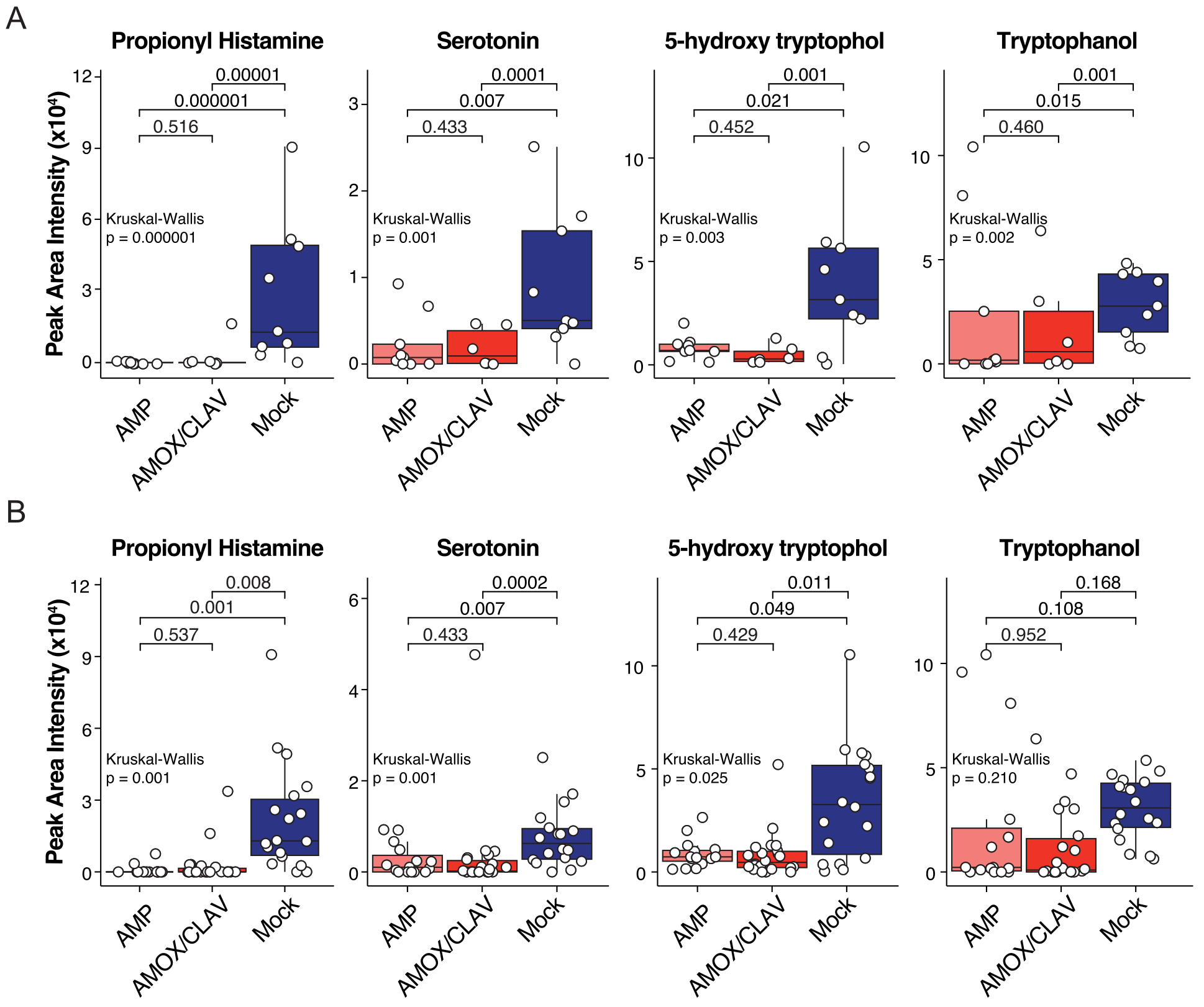
Figure S4. Tryptophan metabolites across treatment conditions in pups. (A)** Univariate analysis was performed on selected molecular features: 5-hydroxy tryptophol (ID 808), serotonin (ID 805), propionyl histamine (ID 481), and tryptophanol (ID 2566). Metabolite levels were compared in pups at Wk2 (pre-sham immunization) through Wk4 (post-sham immunization) following indirect exposure to antibiotics. **(B)** Univariate analysis was performed on selected molecular features: 5-hydroxy tryptophol (ID 808), serotonin (ID 805), propionyl histamine (ID 481), and tryptophanol (ID 2566). Metabolite levels were compared in pups at Wk2 through Wk4 (regardless of immunization status) that were indirectly exposed to antibiotics. Statistical significance (A and B) was assessed using the *Kruskal-Wallis test followed by a Dunn’s test.* The boxplots represent first quartile (lower), interquartile range (IQR), and third (upper) quartile.


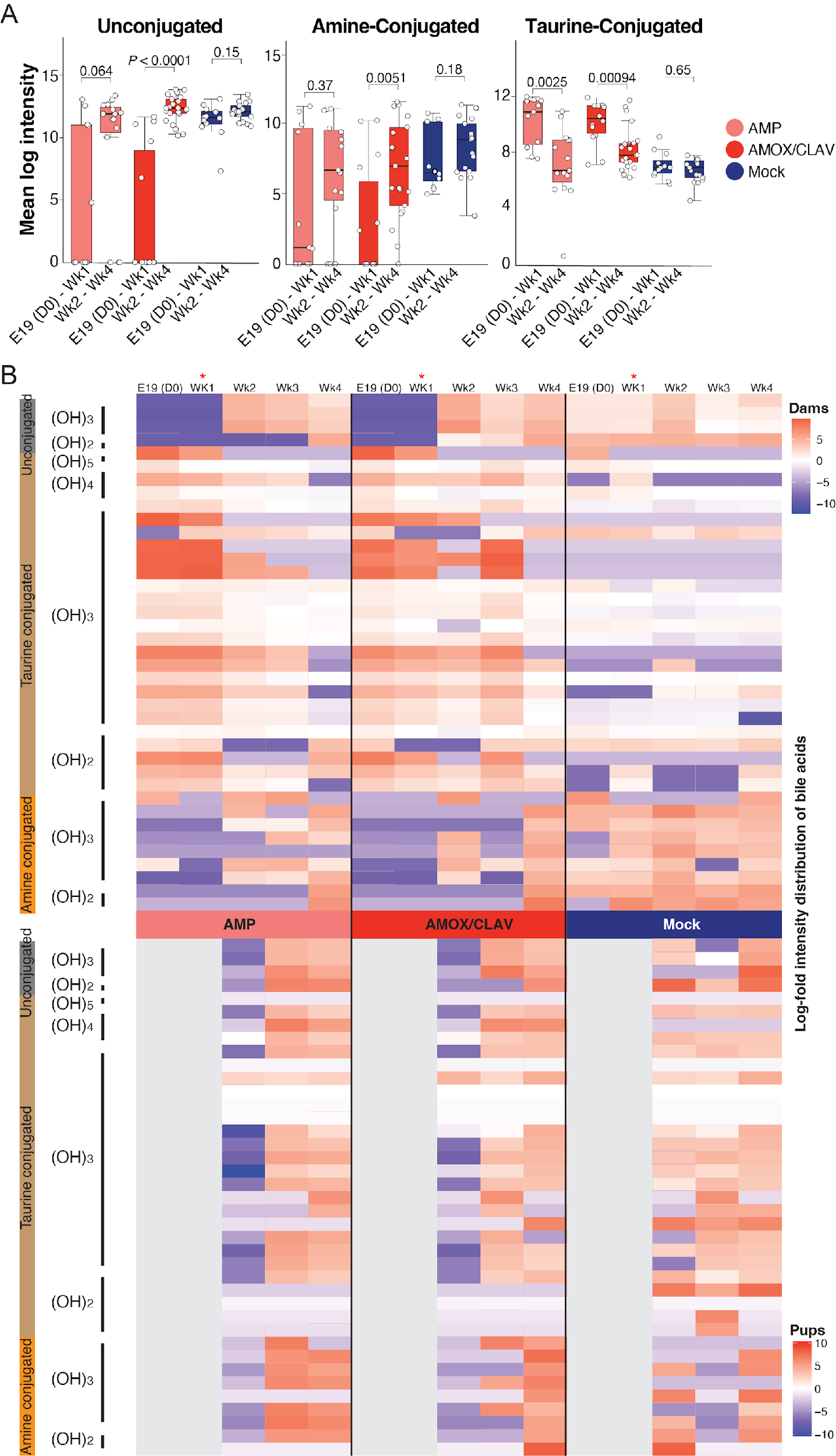


**Figure S5**. **Treatment-specific alterations in bile acid profiles reveal shifts in host and microbial bile acid metabolism.** **(A)** Box plots comparing the mean log intensities of unconjugated, amine-conjugated, and taurine-conjugated bile acids in dams at early (Day 0 to Wk1 postpartum) and late time points (Wk2 to Wk4 postpartum). Feature annotations were obtained from the CMMC-enrichment workflow using GNPS2. **(B)** Heat map of annotated bile acid features showing log-fold intensities across treatment groups in dams and pups (pre- and post-immunized with sham or PCV20). Bile acids are categorized by conjugation type: unconjugated (gray; microbial-derived), amine-conjugated (orange; microbial-derived), and taurine-conjugated (brown; host-derived) spanning diverse hydroxylated core structures. Feature annotations were obtained using the CMMC-enrichment workflow within GNPS2. An asterisk indicates the termination of antibiotic treatment.

**
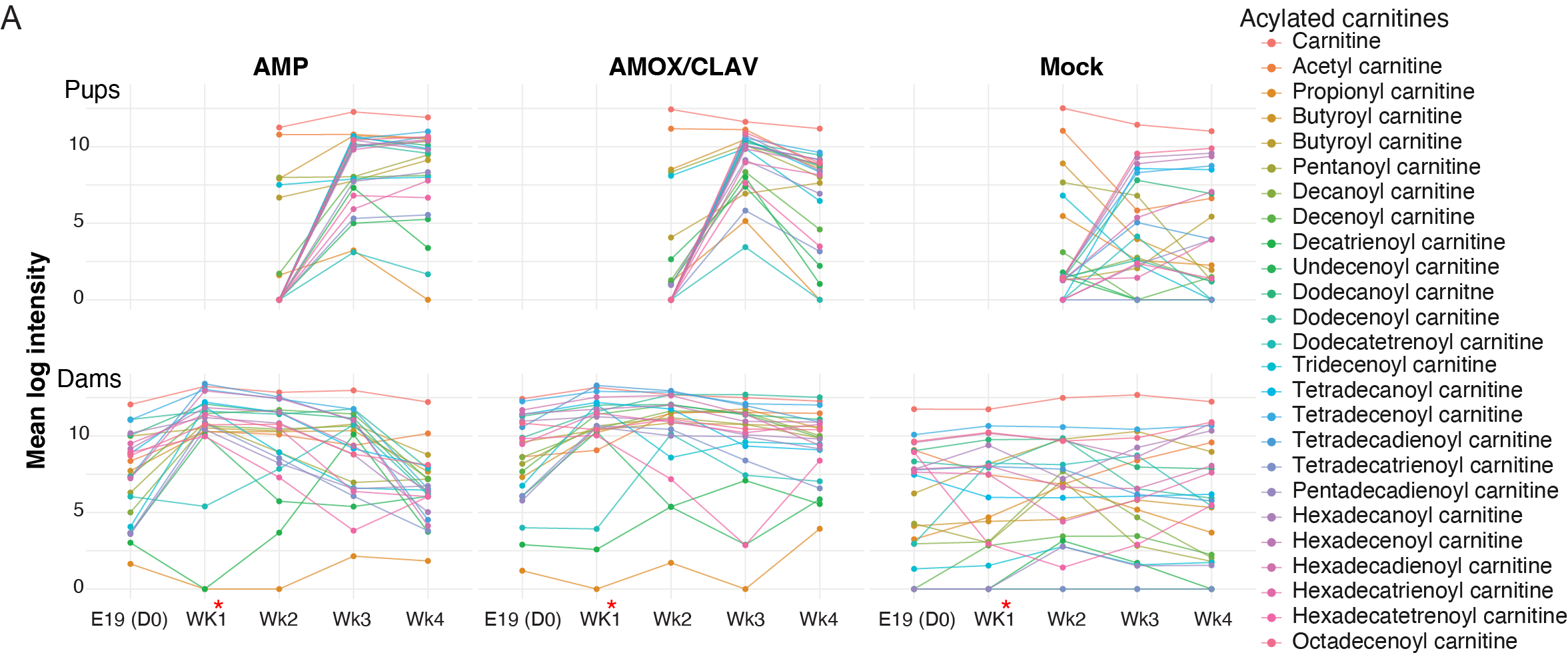
**

**Figure S6**. **Acyl carnitines dynamics across treatment conditions.** **(A)** Line graph depicting the log intensities of MSI level 2-annotated acyl carnitines, a class of host-associated metabolites, across treatment conditions in dams and pups (pre- and post-sham or PCV20 immunization). Feature annotations were obtained using the CMMC-enrichment workflow within GNPS2. An asterisk indicates the termination of antibiotic treatment.

**
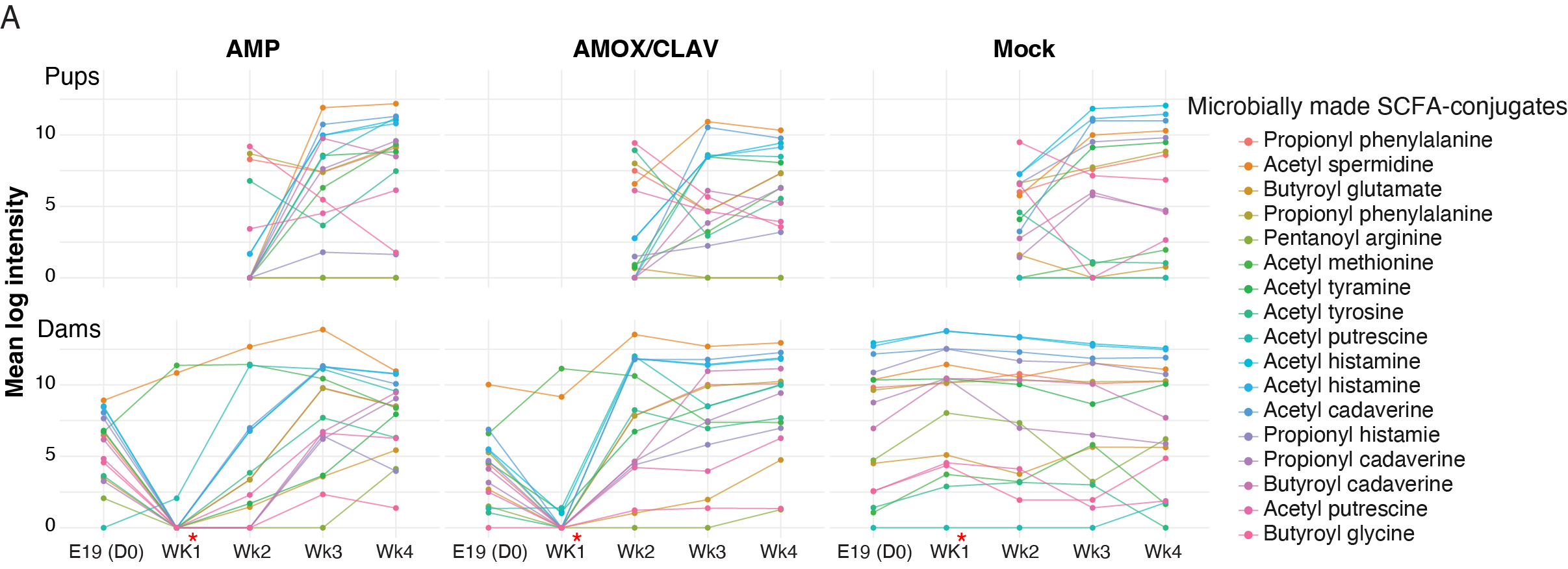
**

**Figure S7**. ***N*-acyl lipids dynamics across treatment conditions.** **(A)** Line graph depicting the log-fold intensities of annotated *N*-acyl lipids, a class of microbe-associated metabolites, across treatment conditions in dams and pups (pre- and post-sham or PCV20 immunization). Feature annotations were obtained using the CMMC-enrichment workflow within GNPS2. An asterisk indicates the termination of antibiotic treatment.

**
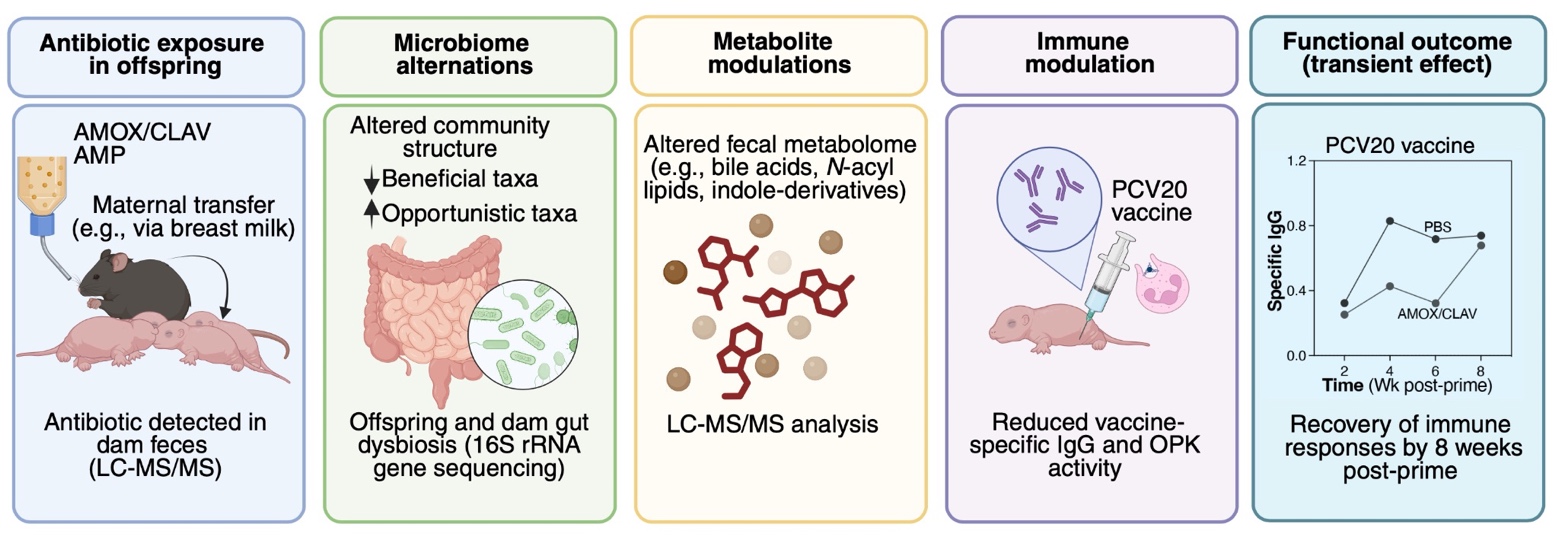
**

**Figure S8**. Schematic overview of the proposed antibiotic-microbiome-metabolite-immune axis underlying early life vaccine responses. The figure was created using Biorender ([Link](https://biorender.com/bb3jz13)).

**Table S1.** LC–MS/MS extracted ion feature abundances in Wk1 postpartum pup serum revealed no detectable features corresponding to AMOX/CLAV or AMP.

| **Sample Category** | **Sample Included** | **AMOX-Related Feature Detected** | **Peak Area Intensity (Range)** |
| --- | --- | --- | --- |
| AMOX (standard)^*^ | AMP.raw | Yes (5/5 features) | 32,304–2,304,572 |
| 6mix (quality control) | 6mix-1 to 6mix-3 | No | 0 |
| Blank | P1-A12; P1-B5 | No | 0 |
| AMP (pups) | P1-A1 to P1-A5 | No | 0 |
| AMOX/CLAV (pups) | P1-A6 to P1-A11 | No^**^ | 0-563 |
| Mock (pups) | P1-B1 to P1-B4 | No | 0 |

^*^A 10 µM pure amoxicillin (AMOX) standard, injected at the end of the sequence, served as a reference.

^**^Single trace signal likely noise
